# Glycogen storage disease type IIIa in pregnant women: A guide to management

**DOI:** 10.1002/jmd2.12282

**Published:** 2022-03-22

**Authors:** Demi Beneru, Michel C. Tchan, Kate Billmore, Roshini Nayyar

**Affiliations:** ^1^ Maternal Fetal Medicine Unit, Department of Obstetrics and Gynaecology Westmead Hospital Sydney Australia; ^2^ Department of Genetic Medicine Westmead Hospital Sydney Australia

**Keywords:** glycogen storage disease type 3, metabolic, obstetric, pregnancy

## Abstract

Glycogen storage disease type IIIa (GSD‐IIIa) is an autosomal recessive disorder that impairs glycogenolysis, producing ketotic hypoglycaemia, hepatomegaly, cardiac and skeletal myopathy. During pregnancy, increased metabolic demand requires careful management. There are few case reports about pregnancy in GSD‐IIIa, however none detail management during caesarean section. This case describes a 25‐year‐old women with GSD‐IIIa diagnosed at 5 months of age. She had modest metabolic control with complications including hepatomegaly, mild skeletal myopathy and poor enteral function requiring multiple operative interventions. She had a planned pregnancy managed by a multidisciplinary team, which included a metabolic geneticist, maternal–fetal medicine specialist and metabolic dietitian. Nocturnal cornstarch was provided to meet basal carbohydrate requirements and a high protein diet with regular carbohydrates was consumed throughout the day. The woman remained well during the antenatal period and had an induction of labour at 38 weeks gestation. She had an emergency caesarean section in early labour due to an abnormal cardiotocography (CTG). The intraoperative and postoperative period were uncomplicated. A live baby boy was born in good condition, weighing 2440 g with APGARs of 9 and 9 at 1 and 5 min. She was managed in labour with glucose 10% IV at 3.5 mg/kg/min, hourly blood sugar level (BSL) monitoring and early epidural anaesthetic. The aim of the first 24‐h post‐partum was prevention of hypoglycaemia, which required strict management with dextrose 10% IV at 3.5 mg/kg/min, oral carbohydrate supplementation and BSL monitoring. This case highlights the complexity of GSD‐IIIa as well as provides a proposed plan for management during pregnancy.

**SYNOPSIS**

This case highlights the complexity of GSD‐IIIa as well as provides a proposed plan for management during pregnancy.

## 1 INTRODUCTION

Glycogen storage disease (GSD) Type IIIa is an autosomal recessive disorder caused by bi‐allelic mutations in the *AGL* gene on chromosome 1p21, resulting in an impaired glycogen debranching enzyme.^1^ This enzyme is important in the breakdown of glycogen. Debranching enzymes normally catalyse the transfer of three glucose moieties to the parent glycogen chain as glycogen breakdown approaches the α‐1,6 branch, as well as subsequent cleavage of the α‐1,6 bond releasing a free glucose molecule. Impairment has two major consequences: ketotic hypoglycaemia and a build‐up of partially broken‐down glycogen, which is then stored in the liver, cardiac muscle and skeletal muscle.^2^ GSD IIIa is characterised by the impairment of the liver, cardiac muscle and skeletal muscle, with this subtype being the most common (affects 85% of GSD Type III individuals).^3^

GSD IIIa typically presents in the first year of life and the common clinical features are hepatomegaly (98%), with associated hypoglycaemia (53%) and failure to thrive (49%).^4^ Patients often undergo extensive investigation before the diagnosis is established. In the past, diagnosis often included a liver biopsy, though more recently multi‐gene panels are often used to confirm the diagnosis. As individuals progress into adolescence and early adulthood hypoglycaemia becomes less of a feature, however myopathy and cardiomyopathy become more prevalent with time.^3^

The principle of management of this condition is optimal dietary control. Children are usually commenced on a high protein diet with regular carbohydrates (usually administered every 3–4 h). In children, nocturnal enteral feeding is often required to meet basal carbohydrate requirements overnight.^4^ In adults, this is usually not required and limited fasting intervals combined with uncooked cornstarch and a high protein diet administered in timed intervals is often sufficient. Uncooked cornstarch (UCCS) is supplemented orally and titrated to maintain normoglycaemia. Regular liver ultrasounds (monitoring for adenomas and fibrosis), echocardiograms (ECGs), blood tests (creatine kinase, liver function tests, and ketones) and consultation with both a biochemical geneticist and dietitian^3^ are required. More recent studies have demonstrated that good dietary control can improve primary manifestations of disease.

There is limited literature with regards to the management of pregnancy in women specifically with GSD Type IIIa, other than a few case reports and one single centre study over a 15‐year period.^5^, ^6^, ^7^ It is now understood that there will be an increased demand for glucose during pregnancy with metabolic requirements increasing in the second and third trimesters. This case report aims to present the management of a woman with GSD Type IIIa, who underwent a caesarean section, resulting in a successful outcome.

## 2 CASE REPORT

A 25‐year‐old woman was diagnosed with GSD IIIa at five‐months of age following an initial presentation with delayed motor milestones. She was subsequently found to have hepatomegaly and the diagnosis was established by liver biopsy. She underwent a percutaneous endoscopic gastrostomy (PEG) insertion at 1 year of age for management of feeding during prolonged hospital admission, and a gastric fundoplication at 4 years of age for management of gastroesophageal reflux.

Her pre‐pregnancy level of creatinine kinase (CK) was significantly elevated (highest level was 2260 U/L) reflecting poor metabolic control. An abdominal ultrasound 5 months prior to conception revealed moderate hepatomegaly, a normal spleen and kidneys, and a transthoracic echo (TTE) performed at the time was normal.

The woman underwent pre‐pregnancy genetic counselling and had a planned conception. Counselling advised that a priori risk of her partner being a carrier for a pathogenic change in *AGL* was around 1 in 160. The parents did not elect to undergo genetic testing to further clarify this risk.

### 2.1 Antenatal care

Her pregnancy was managed by a multidisciplinary team which included a maternal‐fetal medicine specialist, a metabolic geneticist, a high‐risk midwifery nurse consultant and a metabolic dietitian. The antenatal course was uneventful. Routine serology, first trimester ultrasound and morphology scan were within normal limits.

Her pre‐pregnancy weight was 70 kg, and in the first trimester she was prescribed a structured, high protein meal plan with regular carbohydrates, with 90 g (1.3 g/kg ideal body weight) of uncooked cornstarch at 10 pm. Prescribed meals timings were based on the patient's existing meal pattern and were spaced 2–3 h apart. Carbohydrate prescription provided 0.2–0.3 g/kg/h for ideal body weight with an emphasis on not exceeding the prescribed dose to minimise excessive weight gain and the risk of gestational diabetes. The patient was proficient at carbohydrate counting and all carbohydrates throughout the day came from food sources. Protein intake was prescribed at 1.5 g/kg ideal body weight and the patient was given a 7 g protein food exchange list and exchanges were included in her meal plan (Table 1).

**TABLE 1:** Prescribed meal plan for pregnancy based on ideal body weight

Time	Carbohydrate	Protein
8 am	60 g	21–28 g
11 am	30 g	7–14 g
1:30 pm	60 g	21–28 g
4 pm	30 g	7–14 g
6:30 pm	60 g	21–28 g
8:30 pm	30 g	7–14 g
10 pm	90 g uncooked cornstarch (UCCS) (commenced at 14 weeks) reduced to 70 g due to hyperglycaemia at 28 weeks	

There was a significant drop in CK level (from 2260 to 294). Week‐long continuous glucose monitoring was performed six weekly to enable timed adjustments to carbohydrate intake. Weight gain was more than expected by the end of the second trimester, and therefore adjustments in carbohydrate intake were instituted. This included the woman's afternoon and evening carbohydrate dosing, which was reduced due to higher blood sugar levels (BSL) and her nocturnal UCCS was reduced to 70 g. No further dietary changes were required after 20 weeks. When routine antenatal fasting glucose tolerance test (FGTT) was performed at 28 weeks, the woman adjusted the time of the UCCS to 9.5 h before the scheduled time of the glucose test. At 37 weeks, the woman presented for review with a history of decreased fetal movements, and oligohydramnios was noted at the time (amniotic fluid index [AFI] of 7). Induction of labour was planned for 38 weeks gestation, with cardiotocography (CTG) monitoring daily until induction.

### 2.2 Intrapartum care

The woman had the following metabolic intrapartum plan:

#### 2.2.1 Plan during labour

1. Early labour
  - Continue patient's metabolic diet (carbohydrate intake was 30–60 g every 2–3 h) and continue uncooked cornstarch nocturnal 70 g
  - 20% glucose polymers – 200 mL every 2 h
  - 2 hourly BSL monitoring
  - Early epidural anaesthesia
  - Caution with repeated limb occlusion during blood pressure monitoring due to risk of rhabdomyolysis (consider using alternate upper limbs for blood pressure monitoring)
2. Lower segment caesarean section (LSCS)/established labour/not tolerating oral intake
  - Commence IV 10% Glucose at 150 mL/h (3.5 mg/kg/min glucose)
  - Hourly BSL monitoring
  - Early epidural anaesthesia
  - Recommend active third stage (this case received early administration of intramuscular oxytocin with the addition of 40 units of oxytocin in a litre of Hartmann's fluid)

At the time of induction, the woman had a foleys catheter (French 18G) inserted for cervical ripening and underwent artificial rupture of membranes and IV oxytocin the next day. After 5 h of early labour and at 2 cm cervical dilation, the woman had an abnormal CTG (as per NSW Health guidelines, repetitive late decelerations were present).^8^ She proceeded to an emergency LSCS in view of fetal distress in early labour. The LSCS occurred without complication. The estimated blood loss was 300 mL. The live male infant had APGARS of 9 and 9 at 1 min and 5 min. The baby was born in good condition.

### 2.3 Post‐partum care

Routine post‐partum care was provided. In addition, the following metabolic plan was instituted:

#### 2.3.1 24‐hour post‐partum dietary plan

1. First 24 hours post‐partum, if tolerating oral intake
  - Continue patient's metabolic diet; continue UCCS
  - 20% glucose polymers – 200 mL every 2 h
2. First 24 hours post‐partum, if *not* tolerating oral or NG intake
  - Commence IV 10% glucose at 150 mL/h (3.5 mg/kg/min glucose)
  - If BSL > 10 mmol/L, decrease dextrose rate to target 4–8 mmol/L
  - Anti‐emetics as indicated

Post‐partum, she continued following a dietary regime of high protein intake with regular carbohydrate (30–60 g every 3 h), including 70 g nocturnal UCCS. In view of the increased risk of hypoglycaemia in the weeks following delivery due to the increased carbohydrate demands imposed by breastfeeding, 15 g UCCS was recommended prior to each breastfeed. Breastfeeding was ceased around 4 weeks post‐partum (patient's choice). Weekly clinic reviews were undertaken with twice daily blood sugar levels recorded (specifically including morning fasting samples) for 4 weeks. Blood sugar levels were always >4.0 mM. CK levels climbed from 294 to 602 in the first 4 weeks post‐partum (Table 2).

**TABLE 2:** Dietary regime and biochemical picture during each stage

	Dietary regime	Alterations to diet	Biomarkers
Pre‐pregnancy (1 year pre‐partum)	High protein diet at timed intervals, with 70 g UCCS pre‐bedtime	Poor compliance; did not attend dietician regularly	Weight: 70 kg Echocardiogram (ECHO): Normal LV global systolic function with EF 56% Liver ultrasound: enlarged liver (span of 20 cm) Dual energy X‐ray absorptiometry (DEXA) scan: normal bone density CK 2260 ALT 123 AST 156 ALP 99 Random BSL 3.7
During pregnancy	High protein meal plan at timed intervals with regular carbohydrates, and an additional 90 g (1.3 g/kg ideal body weight) uncooked cornstarch at 10 pm (see Table 1)		Weight 83 kg CK 294 ALT 170 AST 259 ALP 128 Random BSL 3.6
Post‐partum	Resumed high protein‐diet with no pre‐bed UCCS but 15 g UCCS every time patient breast feeds. Only continued for 1 week then began formula feeding BSL monitoring 3× per day	After ceasing IV dextrose following caesarean section, commenced 20% glucose polymer drinks (polyjoule) every 2 h for 24 h	Weight 76 kg CK 602 ALT 211 AST 221 ALP 138 Did not attend postpartum ECHO as recommended

## 3 DISCUSSION

This case highlights the importance of careful and early planning in a woman with a metabolic disease. Open communication between the obstetrician, metabolic geneticist and metabolic dietitian were key to creating the management plan. Due to the raised metabolic requirements during pregnancy, there is a significantly increased risk of hypoglycaemia which subsequently may increase the risk of abnormal fetal development or intrauterine death.^7^

Given that the woman successfully met the metabolic requirements of labour, delivery and the post‐partum period without episodes of hypoglycaemia or other crises, this proposed management strategy may be considered in pregnant women with GSD type III.

## CONFLICT OF INTEREST

Demi Beneru, Michel Tchan, Kate Billmore and Roshini Nayyar declare that they have no conflict of interest.

## ETHICS STATEMENT

All procedures followed were in accordance with the ethical standards of the responsible committee on human experimentation (institutional and national) and with the Helsinki Declaration of 1975, as revised in 2000 (5). Informed consent was obtained from the patient included in this article.

## Data Availability

My manuscript has no associated data.
